# Febrile illness and pro-inflammatory cytokines are associated with lower neurodevelopmental scores in Bangladeshi infants living in poverty

**DOI:** 10.1186/1471-2431-14-50

**Published:** 2014-02-18

**Authors:** Nona M Jiang, Fahmida Tofail, Shannon N Moonah, Rebecca J Scharf, Mami Taniuchi, Jennie Z Ma, Jena D Hamadani, Emily S Gurley, Eric R Houpt, Eduardo Azziz-Baumgartner, Rashidul Haque, William A Petri

**Affiliations:** 1 Division of Infectious Diseases and International Health, Department of Medicine, University of Virginia School of Medicine, PO Box 801340, Charlottesville, VA 22908, USA; 2 International Centre for Diarrhoeal Disease Research, Bangladesh, Dhaka, Bangladesh; 3 Division of Developmental Pediatrics, Department of Pediatrics, University of Virginia School of Medicine, Charlottesville, VA, USA; 4 Division of Biostatistics, Department of Public Health Sciences, University of Virginia School of Medicine, Charlottesville, VA, USA; 5 Centers for Disease Control and Prevention, Atlanta, GA, USA

**Keywords:** IL-1β, IL-6, IL-4, Child development, Cognition, Fever, Inflammation, Motor, Neurodevelopment, Pro-inflammatory

## Abstract

**Background:**

An estimated one-third of children younger than 5 years in low- and middle-income countries fail to meet their full developmental potential. The first year of life is a period of critical brain development and is also when most of the morbidity from infection is suffered. We aimed to determine if clinical and biological markers of inflammation in the first year of life predict cognitive, language, and motor outcomes in children living in an urban slum in Bangladesh.

**Methods:**

Children living in Dhaka, Bangladesh were observed from birth until 24 months of age. Febrile illness was used as a clinical marker of inflammation and elevated concentrations of inflammation-related cytokines (IL-1β, IL-6, TNF-α, IL-4, IL-10) in sera collected from a subset of the cohort (N = 127) at 6 months of age were used as biomarkers of inflammation. Psychologists assessed cognitive, language, and motor development using a culturally adapted version of the Bayley Scales of Infant and Toddler Development, Third Edition (Bayley-III) at 12 (N = 398) and 24 months of age (N = 210). We tested for the ability of febrile illness and elevated cytokine levels to predict developmental outcomes, independent of known predictors of stunting, family income, and maternal education.

**Results:**

Every additional 10 days of fever was associated with a 1.9 decrease in language composite score and a 2.1 decrease in motor composite score (p = 0.005 and 0.0002, respectively). Elevated levels of the pro-inflammatory cytokines IL-1β (> 7.06 pg/mL) and IL-6 (> 10.52 pg/mL) were significantly associated with a 4.9 and 4.3 decrease in motor score, respectively. Conversely, an elevated level of the Th-2 cytokine IL-4 (> 0.70 pg/mL) was associated with a 3.6 increase in cognitive score (all p < 0.05).

**Conclusions:**

Clinical and biological markers of inflammation in the first year of life were significantly associated with poor neurodevelopmental outcomes. Conversely, a Th2-like response was associated with a better outcome. These findings suggest that markers of inflammation could serve as prognostic indicators and potentially lead to immune-based therapies to prevent developmental delays in at-risk children.

## Background

An estimated one-third of the world’s children younger than 5 years fail to meet their full developmental potential, increasing the risk that poor health and poverty will follow these disadvantaged children into adulthood, thereby perpetuating the vicious cycles of poverty and impaired development [1,2]. The first year of life is a period of critical and rapid brain development and is also when most of the morbidity and mortality from infection is suffered [1,3]. Recurrent infection in early childhood contributes to stunted growth and development [3,4]. While stunting has been associated with cognitive impairment, mechanisms that result in cerebral damage are not fully understood. It remains controversial whether infection has an independent effect on neurodevelopment through mechanisms such as chronic inflammation [5-7].

The developing brain appears to be particularly vulnerable to inflammatory damage [8]. Infection and inflammation at or near the time of birth have been linked to neurodevelopmental impairments in preterm infants [9-11]. In experimental models, inflammatory cytokines were found to partially mediate inflammation-induced brain damage [9]. Several studies in preterm infants have shown associations of elevated levels of inflammation-related proteins near the time of birth with cognitive dysfunctions years later [12-14]. To the best of our knowledge, no studies have linked markers of inflammation during the post-neonatal period to child development.

Children living in poverty, who suffer disproportionately from chronic and recurrent infections during early childhood, may be at high risk for inflammation-related brain injury. While pneumonia and diarrhea are among the leading causes of infection in children of the developing world, these children also bear a high burden of helminth infections [15,16]. Helminth infections induce a Th-2 immune response in the host, which is characterized by the production of cytokines such as IL-4 [17,18]. The effect of helminth infection on cognitive function in children has been studied, but results across studies are inconsistent and often conflicting [16,19].

We aimed to test whether specific markers of inflammation are associated with developmental outcomes in children. Early identification of at-risk children would allow for the implementation of early and targeted interventions. We hypothesized that both clinical and biological markers of inflammation during the first year of life would be associated with later developmental outcomes. We used febrile illness as a clinical marker of inflammation and the endogenous pyrogens IL-1β, IL-6, and TNF-α as biological markers of inflammation. Additionally, we used IL-4 as a marker of a Th2-like immune response.

## Methods

### Study population and enrollment

This longitudinal study was conducted on a cohort of children living in an urban slum of Mirpur in Dhaka, Bangladesh. Beginning in January 2008, infants were enrolled at birth and followed prospectively. The study period reported here ended in March 2011, when the last child in the study was assessed cognitively at 24 months of age. The study medical officer assessed infants within 72 hours of birth. If the infant was deemed clinically healthy, written informed consent was obtained from the parents or guardians, and the infant was enrolled. The Institutional Review Board at the University of Virginia and the Ethical Review Committee at the International Centre for Diarrhoeal Disease Research, Bangladesh approved this study.

### Active surveillance

Trained field research assistants (FRAs) visited each study household twice a week and collected information on diarrheal disease, respiratory infections, and febrile illness using a structured questionnaire. If a child had an acute illness, he/she was referred to the study clinic to receive medical care. All enrolled children and their family members received free medical services from the study clinic.

### Clinical definitions

Diarrhea was defined as having 3 or more abnormal or unformed stools, as perceived by the mother, in a 24-hour period. Diarrheal episodes were considered distinct if they were separated by at least 3 diarrhea-free days [3]. A child was diagnosed with acute respiratory infection (ARI) if he/she had cough or rhinorrhea. ARI episodes were considered distinct if separated by at least 7 symptom-free days [20]. Fever was defined according to the mother’s subjective assessment, or by axillary temperature of > 37.2°C measured in a household or clinic visit [21].

### Anthropometry

FRAs took anthropometric measurements of each child at the time of enrollment and then every 3 months thereafter. The length of each child was measured to the nearest 0.1 cm (Infantometer Baby Board, Seca 416). Each child was weighed in light clothing on an electronic scale (Digital Baby & Toddler Scales, Seca 354) and the weight was recorded to the nearest 0.01 kg. Length and weight measurements were taken twice and the average of the two measurements was recorded. Anthropometric measurements were converted to length-for-age (LAZ) and weight-for-age (WAZ) scores using WHO Anthro software, version 3.0.1.

### Cytokine measurements

Sera from 127 infants at 6 months of age and were tested for IL-1β, IL-6 TNF-α, IL-4, and IL-10 using the Human Bio-Plex Pro Assays (Bio-Rad, Hercules, CA). Inflammation-related cytokines were measured on every child for which there was 6-month sera available. We chose to test 6-month sera based on the following rationale. First, we wanted to pick an early time point as to enable useful prediction of outcomes, leaving room for potential interventions. Second, we wanted to choose a time at which the majority of children will have suffered at least one infection so that we could discern differences in their cytokine profiles. 12.5 μl of serum was diluted to a 1:3 ratio using the diluent from the kit per the manufacturer’s recommended protocol. The standard positive control included in assay kits was used to generate a standard curve for each target. Standard curves were used to determine the approximate concentrations of all 5 targets for each sample. The Bio-Plex 200 platform was used for detection and Bio-Plex Manager software version 6.0 was used for data analysis.

### Developmental assessment

Trained child psychologists, blinded to the children’s histories and clinical parameters, assessed cognitive, language, and motor development in a clinic setting using a culturally adapted version of the Bayley Scales of Infant and Toddler Development, Third Edition (Bayley-III) [22]. The Bayley Scales have been used by the same research group in several previous studies in rural and urban settings in Bangladesh [23-26]. As the test is mostly non-verbal, the process of cultural adaption focused on modifying pictures in the books while maintaining the original intent of the questions. Field testing of the instrument showed positive correlations of the Bayley scores with child nutritional status and parental education (p < 0.05). Assessment of short-term test-retest reliability (within 7 days) indicated high correlation (r > 0.80). Inter-observer reliability (intraclass correlation) between tester and trainer was high (r = 0.99). Testing was conducted on the children at 12 (N = 398) and 24 (N = 210) months of age. The Bayley-III raw scores for cognitive, language, and motor development were converted to norm-referenced standardized scores (mean = 100, SD = 15) for composite scales [22]. Ten percent of all tests (N = 35) were observed by the supervisor throughout the study period for ongoing reliability.

Approximately 80% of infants enrolled at birth were available for developmental assessment at 12 months of age. Lack of resources prevented us from testing all children at 24 months of age. The children who were and were not tested at 24 months of age did not differ in sex, maternal education, family size, maternal BMI, duration of exclusive breast feeding, or nutritional status at birth. Those who were not tested at 24 months reported higher family incomes and LAZ scores at 12 months, and were less ill in the first year of life.

### Statistical analysis

We evaluated the effect of elevated levels of cytokines and days of febrile illness on neurodevelopment in univariate analysis first. We assessed associations of inflammatory markers with neurodevelopmental outcomes at the initial time of testing (12 months of age) in linear regression analysis, with diagnostics for linearity between predictors and outcome measures, normality, and homoscedasticity. We then evaluated the effect of these inflammatory markers on the repeated measures of developmental outcomes over time using linear mixed effects models. The mixed effects models allowed for evaluation of the change in neurodevelopmental outcomes over one year [27].

Based on the results of the univariate analysis, we further performed multivariable analysis to evaluate the associations of clinical and biological markers of inflammation with neurodevelopmental outcomes after adjusting for baseline characteristics or potential confounders. As many predictors are correlated, such as LAZ and WAZ, we chose only one representative variable from each category we wished to control for. Final multivariable analyses were adjusted for potential confounders of sex, family income, maternal education, and child’s anthropometric status (either 6 or 12 months).

Due to their skewed distributions, cytokine concentrations values were both log-transformed and dichotomized into the highest quartile and lower three quartiles in our initial analyses. In our final models, the cytokine measures were analyzed as binary variables (top quartile v. lower three quartiles) as we were most interested in the contribution of elevated levels of cytokines on neurodevelopment [14]. Statistical significance was defined as a p-value of < 0.05 (two-tailed). Data were analyzed using IBM SPSS 20 (SPSS Inc, Chicago, IL) and SAS 9.3 (SAS Institute, Inc, Cary, NC).

## Results

The children, on average, were malnourished, living in impoverished conditions, and experienced recurrent infection during early childhood (Table 1). The mean household income was < 7000 Bangladeshi taka (BDT) per month (< $90 USD) and nearly 40% of mothers had no formal education. During the first year of life, 398 children experienced 6484 days of diarrhea (16.3 days per child) in 1630 episodes (4.1 episodes per child), 6805 days of acute respiratory infection (17.1 days per child) in 1380 episodes (3.5 episodes per child) and 3845 days of fever (9.7 days per child). Average LAZ and WAZ scores were below average (z-score of 0) at birth and declined over the following 24 months. At birth, 16% of the children were stunted (LAZ < -2). By 24 months of age, the majority of children (60%) were stunted in height.

**Table 1:** Descriptive characteristics of the study population of the total cohort and the subset of children with cytokine profiles

**Characteristic**	**Total (N = 398)**	**Subset (N = 127)**
Male sex (%)	217 (54.5)	74 (58.3)
No maternal education (%)	154 (38.7)	41 (32.3)
Monthly family income (BDT)	6869 ± 3510	7939 ± 4742^*^
Family size	5.5 ± 2.3	5.8 ± 2.9
Maternal BMI < 18.5 (%)	68 (17.1)	18 (14.2)
Exclusive breast feeding (months)	4.0 ± 2.2	3.8 ± 2.3
Low birth weight (%)^†^	131 (32.9)	38 (29.9)
LAZ at birth	-0.95 ± 1.12	-1.04 ± 1.04
LAZ at 12 months	-1.73 ± 1.10	-1.57 ± 1.09
LAZ at 24 months	-2.28 ± 1.08	-2.20 ± 0.78
Courses of antibiotics^‡^	12.2 ± 5.5	13.9 ± 5.3^*^
Diarrheal illness (total days)^‡^	16.3 ± 13.1	12.8 ± 11.1^*^
ARI (total days)^‡^	17.1 ± 13.1	11.6 ± 8.4^*^
Febrile illness (total days)^‡^	9.7 ± 7.8	10.2 ± 7.6

Data are expressed as count (%) for categorical measures and mean ± SD for continuous measures.

Asterisk indicates p-value < 0.05 between children with and without cytokines profiles by t-test for continuous measures and χ^2^ for categorical measures.

*Abbreviations*: *BDT* Bangladeshi taka (currency), *BMI* body mass index, *LAZ* length-for-age Z-score, *ARI* acute respiratory infection.

^†^Less than 2500 g.

^‡^During first year of life.

The mean cognitive, language, and motor composite scores at 12 months of age were 100.3 ± 9.4 (mean ± SD), 98.5 ± 14.0, and 100.7 ± 11.2 respectively, with scores ranging from 65 to 130 for cognition, 62 to 138 for language, and 70 to 145 for motor. At 12 months of age, 0.3% of children exhibited impaired development (score < 70) and 3.5% were affected (score < 85) on the cognition scale, 1.3% were impaired and 18.1% affected on the language scale, and none were impaired and 3.0% affected on the motor scale. The average composite scores in all three domains significantly declined from 12 to 24 months of age. The mean cognitive, language, and motor scores at 24 months were 85.8 ± 8.6, 93.0 ± 10.2, and 96.4 ± 8.5 respectively, with scores ranging from 60 to 115 for cognition, 62 to 121 for language, and 55 to 124 for motor. At 24 months of age, 1.4% of children exhibited impaired development and 39.0% were affected on the cognitive scale, 1.4% were impaired and 17.6% affected on the language scale, and 0.5% were impaired and 3.3% affected on the motor scale.

Univariate analysis using linear mixed models showed that lower birth weight, malnutrition, low maternal education, and poor socioeconomic status were significantly and adversely associated with Bayley-III scores over time (Table 2). For example, each additional kilogram in birth weight increased cognitive, language, and motor scores by 3.1, 3.2, and 3.3 points, respectively. The univariate linear regression results for the baseline variables and predictors of interest are similar to the results from the univariate mixed effects modeling.

**Table 2:** Univariate analysis using linear mixed effects models for repeated developmental outcomes at 12 and 24 months

	**Cognitive composite score**		**Language composite score**		**Motor composite score**	
Bayley-III score at 12 months^*^	100.3 ± 9.4		98.5 ± 14.0		100.7 ± 11.2	
Bayley-III score at 24 months^*^	85.8 ± 8.6		93.0 ± 10.2		96.4 ± 8.5	
**Predictors**	**Estimate (s.e.)**	**p-value**	**Estimate (s.e.)**	**p-value**	**Estimate (s.e.)**	**p-value**
Male sex	-0.79 (0.79)	0.318	-2.22 (1.08)	**0.040**	-2.09 (0.89)	**0.019**
Birth weight (kg)	3.07 (0.94)	**0.001**	3.24 (1.31)	**0.014**	3.33 (1.07)	**0.002**
LAZ at birth (every unit)	1.07 (0.34)	**0.002**	1.32 (0.46)	**0.005**	1.63 (0.38)	**<0.0001**
LAZ at 6 months (every unit)	1.33 (0.38)	**0.0005**	2.22 (0.51)	**<0.0001**	2.12 (0.42)	**<0.0001**
LAZ at 1 year (every unit)	1.41 (0.36)	**0.0001**	2.21 (0.49)	**<0.0001**	2.37 (0.39)	**<0.0001**
WAZ at birth (every unit)	1.40 (0.40)	**0.0005**	1.43 (0.55)	**0.010**	1.56 (0.45)	**0.0006**
WAZ at 6 months (every unit)	1.31 (0.36)	**0.0003**	1.73 (0.50)	**0.0006**	1.77 (0.41)	**<0.0001**
WAZ at 1 year (every unit)	1.64 (0.35)	**<0.0001**	2.35 (0.48)	**<0.0001**	2.40 (0.39)	**<0.0001**
Maternal BMI (every unit)	0.26 (0.12)	**0.038**	0.20 (0.17)	0.258	0.41 (0.14)	**0.004**
Monthly family income (every 1000 taka)	0.38 (0.12)	**0.002**	0.64 (0.16)	**0.0001**	0.57 (0.13)	**<0.0001**
Maternal education (years)	0.25 (0.11)	**0.022**	0.62 (0.15)	**<0.0001**	0.30 (0.13)	**0.019**
Family size (every member)	0.24 (0.17)	0.162	0.21 (0.23)	0.381	0.32 (0.19)	**0.092**
Courses of antibiotics^†^	-0.06 (0.07)	0.388	-0.18 (0.10)	0.070	-0.25 (0.08)	**0.002**
Diarrheal illness (every 10 days)^†^	0.36 (0.03)	0.229	0.47 (0.04)	0.251	0.14 (0.03)	0.678
ARI (every 10 days)^†^	-0.14 (0.03)	0.629	-0.66 (0.04)	0.095	-0.89 (0.03)	**0.007**
Febrile illness (every 10 days)^†^	-1.18 (0.05)	**0.022**	-2.40 (0.07)	**0.0007**	-2.48 (0.06)	**<0.0001**
IL-1β^‡^	1.42 (1.67)	0.398	0.75 (2.38)	0.753	-3.81 (1.71)	**0.028**
IL-6^‡^	-2.94 (1.61)	0.070	-2.85 (2.36)	0.229	-4.64 (1.71)	**0.007**
TNF-α^‡^	0.55 (1.68)	0.746	1.06 (2.40)	0.660	-1.70 (1.80)	0.349
IL-4^‡^	3.38 (1.64)	**0.041**	3.28 (2.34)	0.164	1.21 (1.78)	0.498
IL-10^‡^	2.07 (1.64)	0.209	-0.09 (2.35)	0.971	-0.82 (1.78)	0.648

*Abbreviations*: *BDT* Bangladeshi taka (currency), *LAZ* length-for-age Z-score, *WAZ* weight-for-age Z-score, *BMI* body mass index, *ARI* acute respiratory infection.

^*^Data are expressed as mean ± SD.

^†^During first year of life.

^‡^Each cytokine is dichotomized into top quartile and lower three quartiles, the reference for each cytokine is the lower three quartiles.

Bold text indicates p-value < 0.05.

**Figure 1:**
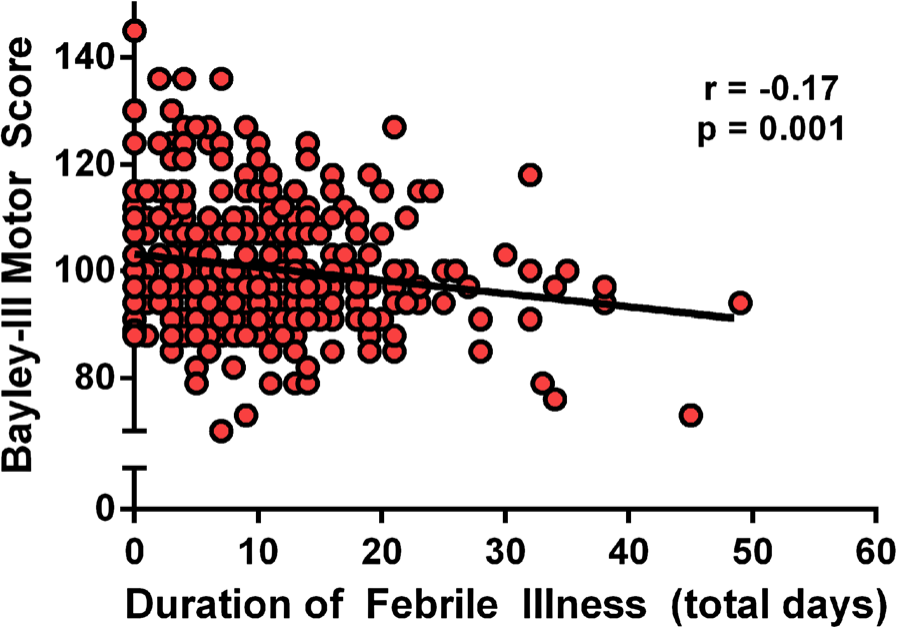
Duration of febrile illness is associated with decreased motor development scores at 12 months.

Linear regression showed that duration of febrile illness in the first year of life was significantly associated with both language and motor development at 12 months of age (Figure 1, language data not shown). Every 10 days of fever in the first year of life correlated with a 1.1 mean reduction in language scores (p = 0.02) and a 1.7 mean reduction in motor scores at 12 months of age (p = 0.001). The association between febrile illness and motor composite score at 12 months remained significant after adjusting for sex, monthly family income, maternal education, and stunting at 12 months of age (β = -1.4, p = 0.005). There remained a trend toward a potential negative association of febrile illness with language composite score after adjusting for confounders (β = -0.8, p = 0.13).

**Figure 2:**
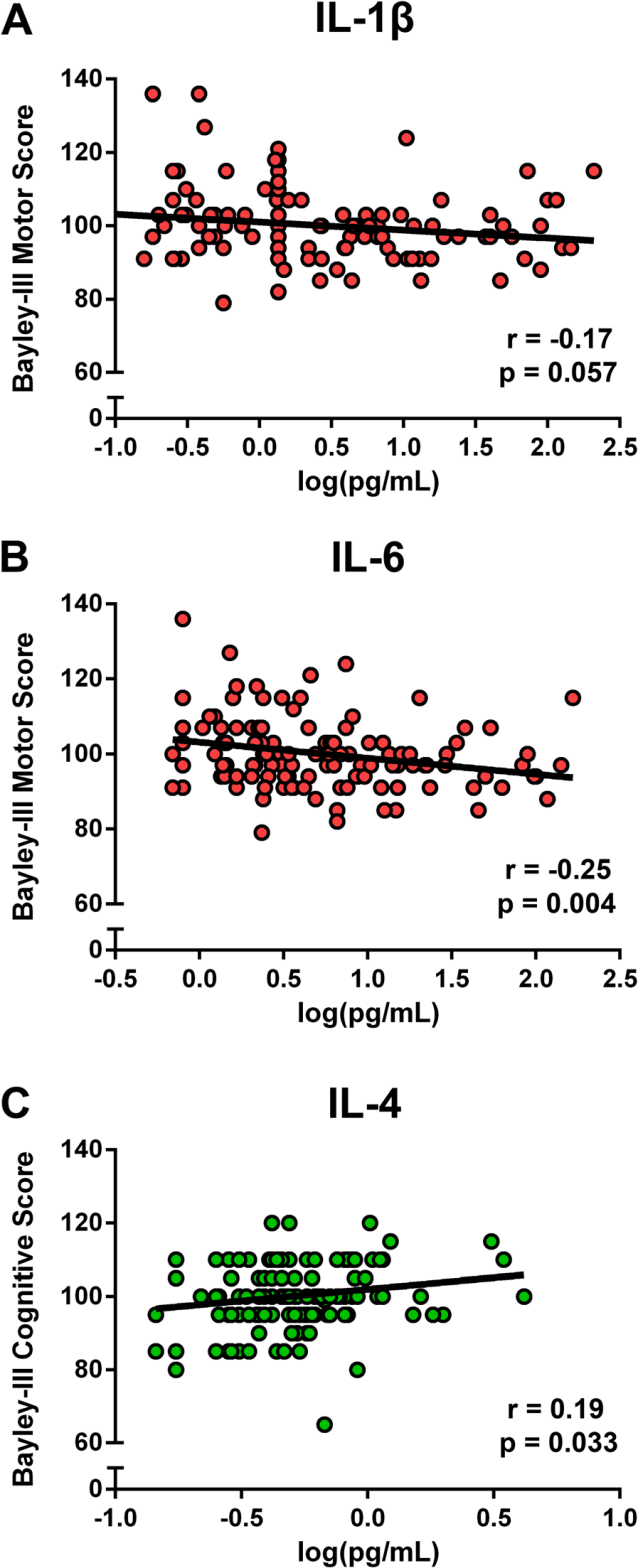
Cytokine levels in 6-month sera are associated with developmental outcomes at 12 months.

Figure 2 shows associations of log-transformed individual cytokine concentrations with developmental outcomes at 12 months of age in a subset of 127 infants. There was a marginally significant trend between IL-1β levels and worse motor outcomes (r = -0.17, p = 0.057). We found a significant association of IL-6 levels with worse motor development at 12 months (r = -0.25, p = 0.004). Conversely, we found a significant association of IL-4 levels with better cognitive development at 12 months of age (r = 0.19, p = 0.033). These associations remained significant after adjusting for sex, monthly family income, maternal education, and stunting at 6 months of age for IL-6 and motor development (β = -0.22, p = 0.011) and IL-4 and cognitive development (β = 0.21, p = 0.020). After the inclusion of either febrile illness or cytokine measures into the final regression model, the incremental improvement in R-square ranged from 0.5 to 4.3%.

The multivariable analysis using mixed models showed a significant association of duration of febrile illness during the first year of life with language and motor development after adjusting for sex, monthly family income, maternal education, and stunting at 12 months of age (Table 3). Every additional 10 days of fever was associated with a 1.9 decrease in language composite score and a 2.1 decrease in motor composite score. Stunting at one year and family income remained significant predictors of language and motor development. Maternal education remained a significant predictor of language development. Cognitive, language, and motor scores increased by approximately 1.1, 1.5, and 2.0 points, respectively for every one unit increment in LAZ score at 12 months of age, and increased by approximately 0.3 to 0.5 points for every 1000 taka increment in monthly family income. Each additional year of maternal education was associated with a 0.4 point increase in language scores.

**Table 3:** Effect of febrile illness on developmental outcomes at 12 months adjusting for sex, monthly family income, maternal education, and LAZ at 12 months

	**Cognitive composite score**		**Language composite score**		**Motor composite score**	
**Predictors**	**Estimate (s.e.)**^**†**^	**p-value**	**Estimate (s.e.)**^**†**^	**p-value**	**Estimate (s.e.)**^**†**^	**p-value**
Male sex	-0.58 (0.77)	0.451	-1.89 (1.02)	0.064	-1.70 (0.83)	**0.041**
Monthly family income (every 1000 taka)	0.25 (0.12)	**0.044**	0.38 (0.17)	**0.026**	0.36 (0.13)	**0.007**
Maternal education (years)	0.10 (0.12)	0.402	0.40 (0.15)	**0.010**	0.02 (0.12)	0.895
Febrile illness (every 10 days)^‡^	-0.88 (0.05)	0.085	-1.91 (0.07)	**0.005**	-2.07 (0.05)	**0.0002**
LAZ at 12 months (every unit)	1.11 (0.37)	**0.003**	1.54 (0.49)	**0.002**	1.99 (0.40)	**<0.0001**

^†^Estimated effect of male sex, monthly family income, maternal education, febrile illness, and LAZ at 12 months on developmental outcomes expressed as the magnitude change in developmental scores for every 1000 taka in family income, 10 days of febrile illness, year of maternal education, and increment in LAZ at 12 months. Data in parentheses are standard errors (SE) for the corresponding covariate effect.

^‡^Days of febrile illness in the first year of life.

Bold text indicates p-value < 0.05.

Table 4 shows the adjusted effects of elevated cytokine concentrations on developmental outcomes. Both elevated IL-1β and IL-6 concentrations were significantly and adversely associated with impaired motor development (p = 0.006 and p = 0.016). Conversely, elevated IL-4 serum levels were significantly and positively associated with higher cognitive scores (p = 0.03). Specifically, children with IL-1β > 7.06 pg/mL had a 4.8 point decrease in motor composite score and those with IL-6 > 10.52 pg/mL had a 4.2 point decrease in motor composite score than those below the cut-offs, respectively. Children with IL-4 > 0.70 pg/mL had a 3.6 point increase in cognitive composite score. While the log-transformed cytokine concentrations were used primarily for our preliminary analyses, they remained significant predictors in the final mixed models just as the binary variables. Income remained a significant predictor of motor development in the models with IL-1β (p = 0.006) and IL-6 (p = 0.01), as well as a significant predictor of cognitive development in the IL-4 model (p = 0.02). Developmental scores increased by approximately 0.3 to 0.5 points for every 1000 taka increment in monthly family income in all three models.

**Table 4:** Effect of elevated cytokine concentrations on repeated developmental outcomes at 12 and 24 months adjusting for sex, monthly family income, maternal education, and LAZ at 6 months

	**Cognitive composite score**		**Language composite score**		**Motor composite score**	
	**Estimate (s.e.)**^**†**^	**p-value**	**Estimate (s.e.)**^**†**^	**p-value**	**Estimate (s.e.)**^**†**^	**p-value**
**Cytokine**^**‡**^						
IL-1β	1.50 (1.72)	0.385	0.41 (2.41)	0.867	-4.88 (1.72)	**0.005**
IL-6	-2.56 (1.66)	0.124	-2.11 (2.34)	0.370	-4.26 (1.73)	**0.016**
TNF-α	0.65 (1.71)	0.704	0.99 (2.38)	0.677	-2.07 (1.81)	0.256
IL-4	3.59 (1.64)	**0.031**	3.21 (2.30)	0.165	0.96 (1.78)	0.592
IL-10	1.75 (1.69)	0.302	-0.85 (2.38)	0.720	-1.50 (1.81)	0.411

^†^Estimated effect for each cytokine expressed as the magnitude change in developmental scores for increment in cytokine category (dichotomized into top quartile and lower three quartiles, reference is the lower three quartiles). Data in parentheses are standard errors (SE) for the corresponding effect.

^‡^Each line represents a distinct mixed effects model for each cytokine measure of interest, adjusting for sex, monthly family income, maternal education, and LAZ at 6 months.

Bold text indicates p-value < 0.05.

The subset of children who were measured for cytokines (N = 127) had similar descriptive characteristics except for income, courses of antibiotics, and illness variables (Table 1). These measures were either directly considered or accounted for through febrile illness in the final analysis. There were 12 children who were febrile for > 30 days, two children who were febrile at the time of sera collection, and seven children who were febrile at the time of neurodevelopmental assessment at either 12 or 24 months. The exclusion of these children did not majorly impact our findings.

## Discussion

The most important finding in this study is that both clinical and biological markers of inflammation independently predict developmental outcomes in a cohort of children from a slum community in Dhaka, Bangladesh. We found that duration of febrile illness and elevated levels of the pro-inflammatory cytokines IL-1β and IL-6 (endogenous pyrogens) in the first year of life are associated with lower neurodevelopmental scores. Interestingly, we also found that elevated levels of IL-4 were conversely associated with higher cognitive scores. To the best of our knowledge, this is the first study to report associations of febrile illness and pro-inflammatory cytokines in the post-neonatal period with developmental outcomes in children. In addition, this study generates the hypothesis that inflammatory (IL-1β and IL-6) and T helper 2 (IL-4) cytokines have opposing effects in infant development.

Our findings that febrile illness and endogenous pyrogens are associated with poor motor development are consistent with the notion that systemic inflammation detrimentally affects the developing brain. Experimental and epidemiologic studies have found that elevated levels of pro-inflammatory cytokines result in white matter damage and cognitive impairment [28-30]. One study in extremely low gestational age newborns reported that elevated levels of IL-1β and IL-6 in the first two weeks of life was associated with an increased risk of cognitive and motor impairments at 24 months of age [14]. Similarly, our study found that elevated levels of IL-1β and IL-6 at 6 months of age were associated with lower motor development scores through 24 months of age. The observation that increased days of febrile illness is correlated with lower developmental scores suggests that persistent or repeated inflammatory insults may have a cumulative effect on developmental outcomes [31].

While the discovery that IL-4 was positively associated with cognitive development was surprising, a beneficial effect of IL-4 on the CNS has support from human studies of the inflammatory disease multiple sclerosis. A first-line treatment for multiple sclerosis (MS) is glatiramer acetate. While the exact mechanism by which glatiramer acetate works is unclear, it is known that the drug induces a Th2 response with IL-4 secretion [32]. In mice models, protection from MS has been achieved with IL-4 delivery to the CNS [33].

Our finding that higher IL-4 levels are associated with better cognition is also consistent with findings in animal models [34]. Mice that lack IL-4 demonstrated cognitive impairment in spatial learning tasks; after transplantation with IL-4 competent bone marrow, this impairment was reversed [34]. A proposed mechanism by which IL-4 promotes cognition is that it exerts its effect through an anti-inflammatory M2-skew of meningeal macrophages, which has been shown to be both beneficial after CNS injury [35] and required for learning [36]. These findings support the concept that a pro-inflammatory state can be detrimental to the developing brain and suggest a potential role for IL-4 in tissue repair and neuroprotection.

One important outstanding question is what is driving the cytokine production in this cohort of children. From the results presented here, it is not possible to conclude if infection is responsible for the IL-1β, IL-6, or IL-4 cytokine production, or if other environmental or genetic differences are responsible for individual differences in cytokine levels. For example, one study found that a rare C variant (instead of G) in the promoter region of the IL-6 gene, which is associated with increased IL-6 synthesis, was associated with impaired cognitive and motor development among children born before 32 weeks gestation [37].

The microbiome may also contribute to systemic pro- (IL-1β or IL-6) or anti-inflammatory (IL-4) immune responses. Components of the microbiota are known to influence circulating levels of cytokines in both humans and mice, which may in turn affect the CNS [38,39]. Specific components of the microbiota may directly drive cytokine production by immune cells. Alternatively, metabolic products produced by the microbiota may also influence differences in serum cytokine levels [39].

A key limitation of our work is that we were not able to obtain cytokine profiles on the full cohort in this exploratory study. In addition, cytokine profiles were only obtained at one time point (6 months) and not throughout the 24 month period of observation. The impact of systemic cytokines on development throughout the critical first few years of life remains to be fully understood. Until further studies have been conducted that compare the predictive ability of cytokines in sera collected at multiple time points, our data should not be extrapolated to other time points. Another main limitation of our study is that we were not able to conduct neurodevelopmental assessments on the entire cohort at 24 months of age. It should be noted, however, that our major findings were significant even when only considering data at 12 months of age. Furthermore, our data on febrile illness partially relied on the mothers’ subjective assessment of fever. However, studies from several countries have consistently reported that mothers are able to accurately assess the presence of fever in their children [40-43]. Finally, while we were able to adjust for family or environmental factors known to impact child development, there remain other potential confounding factors, such as maternal depression, that were not measured in this study [44].

There is arguably no greater problem than impaired child development, which affects millions of the world’s poorest and most vulnerable children. The first few years of life are the most crucial in a child’s development as they lay the groundwork for emotional, physical, and intellectual wellbeing [1,2]. This study generates important questions about the role of infection and inflammation in child development, which merit further investigation. Our work has identified immune correlates of developmental outcomes in children. These correlates could potentially serve as prognostic markers or lead to new immune-based therapies to prevent developmental delay in at-risk children and to avoid unaffordable losses in human potential.

## Conclusions

We found that duration of febrile illness and elevated levels of the pro-inflammatory cytokines IL-1β and IL-6 in the first year of life were associated with poor developmental outcomes. Elevated levels of the Th2 cytokine IL-4 were conversely associated with higher cognitive scores. Further studies are urgently needed to validate these important epidemiological findings and to elucidate the underlying cause of elevated cytokine levels.

## Abbreviations

ARI: Acute respiratory infection; Bayley-III: Bayley Scales of Infant and Toddler Development, Third Edition; BDT: Bangladeshi taka; BMI: Body mass index; CNS: Central nervous system; IL: Interleukin; LAZ: Length-for-age Z-score; TNF: Tumor necrosis factor; WAZ: Weight-for-age Z-score.

## Competing interests

The authors declare that they have no competing interests.

## Authors’ contributions

NMJ directed the research, conducted the data analysis, and drafted the manuscript. SNM, RJS, JZM, and ESG assisted in the data analysis and manuscript preparation. FT and JDH organized the cognitive testing of the children and the collection of the developmental data. MT and ERH developed and performed the cytokine assays. EA directed the collection of the respiratory data. RH directed the Bangladesh site. WAP conceived the idea and coordinated the work. All authors have seen and approved the final manuscript.

## Pre-publication history

The pre-publication history for this paper can be accessed here:

http://www.biomedcentral.com/1471-2431/14/50/prepub
